# Linking Environmental Exposure with Public Health: Dichlorodiphenyltrichloroethane Extracted from Soils and Water of Recently Exposed Communities of Selected Locations in Zambia

**DOI:** 10.1155/2015/564189

**Published:** 2015-10-22

**Authors:** Nosiku Sipilanyambe Munyinda, Charles Michelo, Kwenga Sichilongo

**Affiliations:** ^1^Department of Public Health, Environmental Health Unit, School of Medicine, University of Zambia, Zambia; ^2^Department of Chemistry, Faculty of Science, University of Botswana, Gaborone, Botswana

## Abstract

*Background*. In 2000, a Zambian private mining company reintroduced the use of dichlorodiphenyltrichloroethane (DDT) to control malaria in two districts. From 2000 to 2010, DDT had been applied in homes without any studies conducted to ascertain its fate in the environment. We aimed to quantify the presence of DDT and its metabolites in the soil and water around communities where it was recently used. *Methods*. We collected superficial soil and water samples from drinking sources of three study areas. DDT was extracted by QuEChERS method and solid phase extraction for soils and water, respectively. Analysis was by gas chromatography-mass spectrometry. A revalidated method with limits of detection ranging from 0.034 to 0.04 ppb was used. *Results*. Median levels of total DDT were found at 100.4 (IQR 90.9–110) and 725.4 ng/L (IQR 540–774.5) for soils and water, respectively. No DDT above detection limits was detected in the reference area. These results are clinically significant given the persistent characteristics of DDT. *Conclusion*. DDT presence in these media suggests possible limitations in the environmental safeguards during IRS. Such occurrence could have potential effects on humans, especially children; hence, there is a need to further examine possible associations between this exposure and humans.

## 1. Introduction

Present benefits versus discounted costs? This question is still unanswered in terms of the use of DDT (dichlorodiphenyltrichloroethane), a persistent organic pollutant used for malaria control in many parts of Africa. In Zambia, malaria is still the leading cause of morbidity and mortality accounting for 36% of all hospital admissions with the majority being pregnant women and children [1]. In response to this high malaria burden, the government of Zambia through the Ministry of Health developed an integrated vector management (IVM) strategy [1, 2]. This strategy focused on increasing coverage of indoor residual spraying (IRS) activities in addition to distribution of insecticide-treated nets (ITNs), expansion of environmental management, and larviciding. In 2000, a private mining company, Konkola Copper Mines, was the first to reintroduce DDT as one of its main chemicals for IRS after more than two decades since its use had been discontinued in Zambia [3]. This followed the total ban of DDT in the United States of America and other developed countries in the early 1970s due to its negative environmental effects [4]. These IRS activities were complemented by other IVM interventions. The apparent success of this programme in one mining town and the fact that IRS with DDT was the principal method by which malaria was eradicated or in some cases significantly reduced in many countries in the world led the Zambian government in 2003 to initiate IRS activities initially in five pilot districts [1, 5]. Several World Health Organisation- (WHO-) endorsed insecticides, including DDT, which was applied only to unplastered surfaces, were used for this activity. This initial coverage was increased to 15 districts in 2008.

Several studies have shown that DDT causes thinning of bird egg shells and finds its way into the food chain if improperly handled [6–8]. It is an endocrine disruptor and has been shown to cause other human health effects ranging from reproductive failure to increased incidences of different cancers [4, 9–25]. Its main action is through the disruption of the neurological functions of the brain by disturbing neurotransmitters, mimicking Th4, a thyroid hormone, and disturbing the internal signalling system of synapses [21, 25–35].

Locally, 239,758 kg of DDT has been used in Zambia since 2000 with no documented studies being conducted on environmental and human exposures [36]. Previous studies on DDT exposure in other settings involved historically exposed communities and looked mainly at its metabolites DDE and DDD. The few studies done in South Africa focused on reproductive outcomes in occupationally exposed males and some in women [9, 15]. Given the potential harm that DDT has been shown to cause in various locations around the world, coupled with the HIV pandemic and poverty situation in Zambia, it is highly likely that these effects will be more pronounced in the Zambian population. So far, no study has been carried out to ascertain the fate of the recently applied DDT in both the environment and human health.

To this effect, we aimed to carry out an exposure assessment in selected areas of Zambia in order to quantify the prevalence of DDT and its metabolites in soil and water around communities where it was recently used. This work is part of a broader research project which aims to link recent DDT exposure to neurodevelopmental outcomes in children of the targeted populations and also evaluate other factors that may be associated with the observed outcomes.

## 2. Materials and Methods

### 2.1. Study Areas

The study areas comprised Chawama, Chongwe, and Mongu, selected based on history of past exposure to DDT in the last three years. Chawama was randomly selected for this study as it was one of the pilot areas where DDT was applied in 2004 and has a very high malaria burden compared to the rest of Lusaka. It is a periurban area in the center of Lusaka situated at 15.35° south latitude, 28.7° east longitude, and 1069 meters above the sea level. Chongwe, on the other hand, is a rural town east of Lusaka situated at 15.35° south latitude, 28.7° east longitude, and 1069 meters above the sea level. It was also randomly selected among among two other areas, Kafue and Mumbwa, which are rural districts within a 200 km radius to Lusaka, as it was one of the areas where DDT was applied commencing in 2008. In contrast, Mongu is a town with a mixed urban and rural population located west of Lusaka situated at 15.25° south latitude, 23.13° east longitude, and 1018 meters above the sea level. It was conveniently selected as the control area because there is no report of DDT application as part of government IRS activities [37]. Mongu was selected as the reference area while Chawama and Chongwe are the areas where DDT was applied.

### 2.2. Sampling

The sampling for soil and water in all the study areas was done during July 2012. Compound samples were collected from locations in the immediate periphery of the houses.

The inclusion criterion for the areas with recent application was that the residences should have been sprayed at least three times with DDT in the last 10 years.

#### 2.2.1. Soil

**Figure 1:**
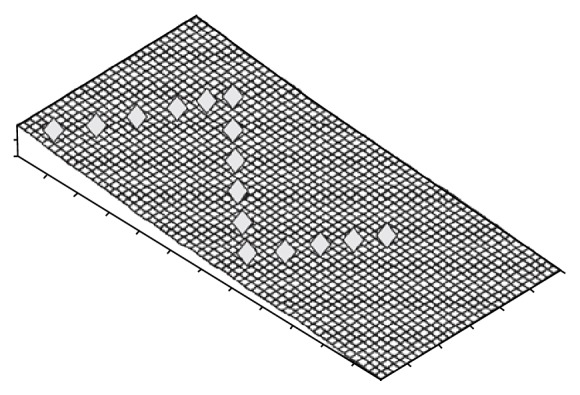
Soil sampling pattern.

A total of 14 soil samples were collected in Chongwe (3), Chawama (7), and Mongu (4). In order to discern recent exposure characteristics, samples were obtained from the top soil at a depth of 2–5 cm using bucket and tube augers depending on the type of soil present in the area. At each sampling point, 500 g of the soil sample was collected and placed in a nontransparent paper bag which was put into a plastic bag to avoid leakage. In order to ensure representativeness of the soil samples in the study areas, a zigzag sampling pattern was employed covering the entire sampling area in each community as illustrated in Figure 1.

#### 2.2.2. Water

A total of 14 water samples were collected in Chongwe (3), Chawama (7), and Mongu (4), respectively. The samples were obtained from various drinking water sources such as shallow wells, open streams, and communal taps. These samples were obtained using 1-litre water sample bottles which had been triple-rinsed with wash grade acetone/hexane mixture. The bottles had tightly fitted caps and lids to avoid spillages and enough space was allowed at the neck of the bottle to facilitate air exchange.

The samples were transported to the Mass Spectrometry Unit in the Department of Chemistry at the University of Botswana under the United Nations Transport of Dangerous Goods (UNTDG) Regulations of 2011 [38] where they were kept in a cold room at ~4°C until they were extracted and analysed.

### 2.3. Sample Extraction and Chemical Analysis

#### 2.3.1. Soil Samples

A slightly modified AOAC Method 2007.01 adapted by Nagel [39, 40] using QuEChERS kits was used for extraction of DDT from soil samples. Twenty (20) grams of soil was weighed into a centrifuge tube, and 12 mL of water was added and shaken for 4 hours after which 20 mL of acetonitrile was added for homogenization. The supernatant was thereafter quantitatively transferred to a second centrifuge tube containing 6 g MgSO_4_, 1.5 g NaCl, 1.5 g Na_3_Citrate dihydrate, and 750 mg Na_2_HCitrate sesquihydrate. Centrifugation was done for 2 minutes at 4500 rpm. Sample cleanup was achieved by using dispersive solid phase extraction using cartridges that contained 150 mg primary secondary amine (PSA), 400 mg C18EC, and 900 mg magnesium sulfate of between 98.5 and 101.5% purity. In each extraction, 6 mL of supernatant was transferred into an SPE cartridge and shaken for 1 minute before centrifugation for 2 minutes at 4500 rpm. The organic layer was filtered using a syringe and a membrane filter (0.45 mm) and then evaporated under nitrogen to 1 mL. The concentrate was transferred into a 2 mLGC vial and was ready for gas chromatography-mass spectrometry (GC-MS) analysis.

#### 2.3.2. Water Samples

The standard solid phase extraction (SPE) method of conditioning, loading, washing, and elution was used for the extraction of DDT from the water samples. This was achieved using Florisil SPE Cartridges and an Agilent vacuum manifold (*VacElut SPS 24*). The procedure was a slight modification of a method developed in the laboratory for the determination of PCBs in transformer oil [41]. Eight (8) millilitres of hexane was added to condition the cartridge followed by 8 mL of deionised water at 1 mL/min. This was followed by loading 20 mL of the water sample at a flow rate of 0.5 mL/min. The cartridge was allowed to dry for another 20 minutes to allow for interaction of the DDT with the stationary phase.

Five (5) millilitres of hexane was eluted through the cartridge to wash off any nonpolar analytes that could have bound strongly to the stationary phase. Elution of the DDT was accomplished by running 10 mL of a 5 : 5 n-hexane : acetone mixture. The resulting eluate was blown gently under a stream of nitrogen to concentrate it to 0.5 mL and transferred to GC vial for GC-MS analysis.

### 2.4. GC-MS Conditions

An Agilent (Palo Alto, CA, USA) 5975 C series gas chromatograph-quadrupole mass spectrometer (GC-qMS) system equipped with a selective mass detector was used for analysis. The system has capability to perform ion manipulation in the full scan and selected ion monitoring (SIM) modes. It also has two ionization modes, that is, electron ionization (EI) and chemical ionization (CI). In this study, the SIM mode was used after CI using methane as a reagent gas. Helium was used throughout as a carrier gas at a flow rate of 1 mL/min. The oven program for the GC was as follows: The column was held at 80°C for 2 min and then ramped at 10°C/min up to 270°C for a total run time of approximately 30 minutes. The injector was held at 270°C. The mass spectrometer was operated in the negative chemical ionization (NCI) mode for soil analysis while the electron ionization (EI) mode was used for water sample extracts. All the instrumental settings used were optimized prior to application on real sample extracts. The Automated Mass Spectral Deconvolution and Identification System (AMDIS) by the National Institute for Standards and Technology (NIST) in conjunction with Microsoft Excel was used for data analysis. Extraction efficiencies were determined by analysis of spiked sample extracts with the analytes of interest.

### 2.5. Quality Control

For quality control and assurance, procedural blanks were extracted with n-hexane and subjected to the same extraction procedure as the other samples. These were run with every ten samples in addition to field and laboratory blanks. Descriptive statistics were used to describe data and the term tDDT was used to refer to the sum of all DDT and its metabolites DDE and DDD. In the presentation of the results, however, a distinction was made as to which DDT metabolite was being referred to.

### 2.6. Ethics

Written permits were acquired from the Ministry of Mines and Mineral Resources to export the soils and water into Botswana for analysis. The Botswana Unified Revenue Service also gave entry permission for the samples.

## 3. Results

### 3.1. DDT Distribution and Validation

A summary of validation results derived from 6 point calibration curves with corresponding regression equations is presented in Table 1.

From Table 1, reasonably good extraction efficiencies were attained using both QuEChERS and SPE extractions. Low detection limits were also estimated in both soil and water samples and when these are compared to the 1 *μ*g/L which is the World Health Organization (WHO) Maximum Recommended Limit (MRL) for DDT and its derivatives in drinking water, they were found to be low enough for analysis.

**Figure 2:**
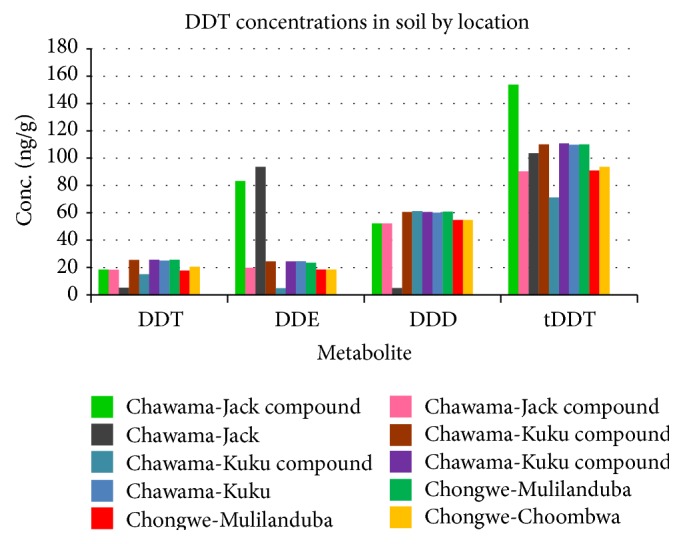
DDT, DDE, and DDD concentrations in soil samples from Chawama and Chongwe.

Results of concentrations of DDT and its metabolites in the study areas are summarized in Figure 2.

Figure 2 shows that all analytes were present in all the soils of Chawama and Chongwe. However, they were all found to be below the LoDs in Mongu which was our reference area. The concentrations varied in all the study areas but the highest amounts of DDT at 25.8 ng/g were determined around a rural homestead of Chongwe followed by 25.7 ng/g in soils collected very close to the exterior walls of a house in Chawama Township. DDT accounted for 12% of the tDDT in the soils sampled. In some areas, the percentage was as high as 27%.

**Figure 3:**
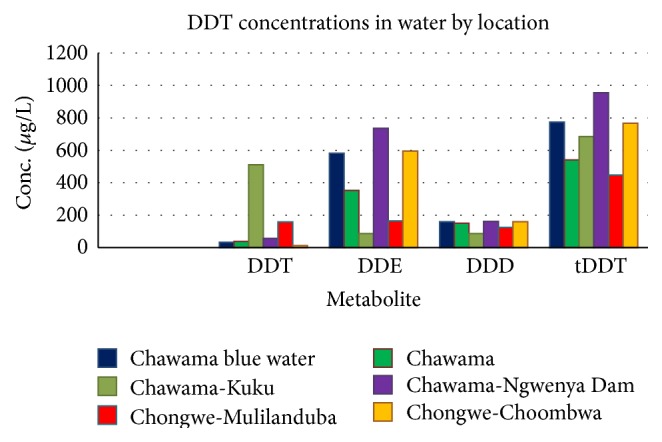
DDT, DDE, and DDD concentrations in water samples from Chawama and Chongwe.

In water, the sample analysis results are shown in Figure 3.

DDT in water was found in 6 out of the 14 sampling points. No DDT above the limits of detection was detected in water samples from Mongu, which was the reference sampling site. In general, the lowest amount of DDT accounted for 4% of the tDDT while the highest concentration at 511 ng/g was detected around a pit latrine in Chawama Township translating into 75% of tDDT.

## 4. Discussion

DDT levels were found in significant levels in both water and soils in the study areas of Zambia. These high levels were most prominent in sites with a history of DDT exposure through the IRS program for malaria control. No DDT above the limits of detection was found in the reference area. In soil from the two Zambian study areas, the median concentrations were twice or higher than those reported in Spain, Uganda, and South Africa among selected countries where they were 46, 59, and 43 ng/g, respectively [42–44].

It is possible that there might be inherent sampling errors and that, coincidentally, both sampled sites with recent exposure could have higher DDT concentrations as found in this study. However, given that every effort was made to randomly select the locations and sampling sites, it is unlikely that these sampling errors could be important enough to explain these results. We therefore reasonably argue that what was found in this exposure assessment could be from recent exposure.

DDT has not been sprayed in Zambia since 2010 when evidence of mosquito resistance to it began to emerge [45]. DDT and its metabolites can persist in soil for 2–15 years [8] and therefore the period of recent exposure still falls within the half-life brackets for DDT. The soil sampling protocol restricted the depth at which the soil samples were collected to the A and E horizons which are generally about 0.6–4.5 m below the surface and typically lose minerals and chemicals due to leaching over time [46]. The study conducted in Kenya by Lalah et al. showed that DDT and other pesticides metabolize faster at varying rates depending on the soil type due to environmental factors such as prevailing climatic conditions, pH of the soil, and the action of microorganisms. It has been shown to degrade even faster in temperate climates such as the one found in Zambia.

DDT has a much longer half-life of up to 150 years in water, and due to its lipophilic nature it tends to gravitate towards organic material and other such fatty tissues. Its very high concentrations in the water bodies where the samples were collected were a surprising and alarming result. The median concentrations of DDT were found to be more than two hundred times higher than those recorded in Nigeria and South Africa at less than 0.368 and 2 *μ*g/L, respectively. This is against the background in which the WHO has recommended a maximum of 1 *μ*g/L per 0.01 mg/kg of body weight calculated at the assumption that a 10 kg child drinks up to 1 litre per day [47]. These results corroborate the findings from the 2010 Environmental Council of Zambia (ECZ) audit of IRS activities which showed lapses in the implementation of environmental safeguards during the spraying exercises [36].

No DDT and its metabolites above detectable limits were detected in the reference area in both soil and water samples. Mongu was not included in the IRS program due to its proximity to the Zambezi River, a source of livelihood and nutrition for the local communities. Despite the historical application of DDT in the 1940s to the 1980s for other purposes in Mongu, the sampling of only elluvium A top soil could have masked residues which most likely may be present in the lower strata. Given the rapid velocity of the water in the Zambezi River and the high water table in Mongu, it is highly unlikely that DDT could have remained in the aquatic system. This coupled with the presence of many aquatic species and other organic materials in the water bodies in this area could have resulted in the DDT sequestering itself in them due to its lipophilicity.

These results are clinically significant given the bioaccumulation and biomagnification characteristics of DDT as it travels up the food chain [7, 8, 47, 48]. Several studies in various settings of the world have shown that DDT in plants is taken up through the roots and when these plants are consumed by both humans and animals, the DDT remains sequestered in these species' adipose tissue. This is also true when DDT sequesters itself in aquatic species which are edible to humans and animals. These studies also show that the primary exposure route for humans to DDT is through ingestion of contaminated foods and water [47].

The high tDDT concentrations found in the study sites and subsequent DDT contamination burden may be an indication of challenges associated with environmental monitoring of such pollutants especially in resource poor settings already plagued with high malaria incidences among other public health challenges (Table 2). Given that these highly exposed areas are also often inhabited by very poor populations most of whom are women and children, this poses an ethical dilemma to decision makers on the cost-effectiveness of reintroducing DDT. Speculations were raised by several scholars on the effectiveness of the reintroduction of DDT for malaria control. The researchers in [10, 49, 50] are thus justified. This is based on results of studies such as this one and that conducted in South Africa also which showed that DDT had contaminated the soils, water, and livestock of previous IRS communities [42].

## 5. Conclusions

The presence of DDT and its metabolites in environmental samples from soils and water of selected study areas has been demonstrated. Given that the breakdown products DDE and DDD are more stable in the environment and human matrices and have been implicated in dire effects, urgent action is required. This calls for more investment in surveillance and environmental monitoring in order to develop effective remediation solutions that will rapidly break down this DDT and thereby remove it from the food chain. Furthermore, the cradle-to-grave principle of waste management must be applied to this dilemma as the cost of DDT reintroduction is currently being borne by the public and given the persistent nature of DDT even unborn children will suffer the consequences of this lapse in environmental stewardship. Hard choices driven by appropriate leadership may have to be made which favour a win-win situation for current and future generations.

## Figures and Tables

**Table 1:** Method validation parameters.

Metabolite	Soil				Water			
	Regression equations and *R* ^2^ values	Retention time (min)	LoD, ng/kg (ppb)	% recovery	Regression equations and *R* ^2^ values	Retention time (min)	LoD, *µ*g/L (ppb)	% recovery
DDT	*Y* = −0.155*x* ^2^ *x* ^2^ ^2^ + 1.927*x* + 4.200 *R* ^2^ = 0.978	20.140	0.046	86.8	*Y* = −0.039*x* + 0.380 *R* ^2^ = 0.962	20.140	0.000027	79.2

DDE	*Y* = *y* = −0.155*x* ^2^ *x* ^2^ ^2^ + 1.928*x* + 4.177 *R* ^2^ = 0.978	17.435	0.040	88.4	*y* = 0.104*x* + 0.036 *R* ^2^ = 0.992	17.435	0.000009	79.9

DDD	*y* = −0.242*x* ^2^ *x* ^2^ ^2^ + 3.143*x* + 3.199 *R* ^2^ = 0.992	17.786	0.034	91.6	*y* = −2.644*x* + 9.211 *R* ^2^ = 0.991	17.786	0.000008	83.2

**Table 2:** Summary description of tDDT concentrations in Chawama and Chongwe.

	Soils (Chongwe), ng/g	Water (Chongwe), *µ*g/L	Soils (Chawama), ng/g	Water (Chawama), *µ*g/L
Mean	98.1	404.7	106.9	405.1
Min	90.9	bdl^*∗*^	71.1	bdl
Max	109.8	766.9	154.4	971.5
Median	93.6	447.2	107.2	341.9

^*∗*^Below detectable limits.
